# Safety Assessment of Casting Workshop by Cloud Model and Cause and Effect–LOPA to Protect Employee Health

**DOI:** 10.3390/ijerph17072555

**Published:** 2020-04-08

**Authors:** Qingwei Xu, Kaili Xu, Fang Zhou

**Affiliations:** 1 College of Information and Management Science, Henan Agricultural University, Zhengzhou 450046, China; 2 Key Laboratory of Ministry of Education on Safe Mining of Deep Metal Mines, School of Resources and Civil Engineering, Northeastern University, Shenyang 110819, China; xukaili@mail.neu.edu.cn

**Keywords:** safety assessment, employee health, cloud model, cause and effect–LOPA, least square method

## Abstract

Safety assessment of a casting workshop will provide a clearer understanding of the important safety level required for a foundry. The main purpose of this study was to construct a composite safety assessment method to protect employee health using the cloud model and cause and effect–Layer of Protection Analysis (LOPA). In this study, the weights of evaluation indicators were determined using the subjective analytic hierarchy process and objective entropy weight method respectively. Then, to obtain the preference coefficient of the integrated weight more precisely, a new algorithm was proposed based on the least square method. Next, the safety level of the casting workshop was presented based on the qualitative and quantitative analysis of the cloud model, which realized the uncertainty conversion between qualitative concepts and their corresponding quantitative values, as well as taking the fuzziness and randomness into account; the validity of cloud model evaluation was validated by grey relational analysis. In addition, cause and effect was used to proactively identify factors that may lead to accidents. LOPA was used to correlate corresponding safety measures to the identified risk factors. 6 causes and 19 sub-causes that may contribute to accidents were identified, and 18 potential remedies, or independent protection layers (IPLs), were described as ways to protect employee health in foundry operations. A mechanical manufacturing business in Hunan, China was considered as a case study to demonstrate the applicability and benefits of the proposed safety assessment approach.

## 1. Introduction

In recent decades, China’s economy has developed rapidly and citizens’ living standards have greatly improved [1,2,3]. With continued economic growth, China’s foundry industry has achieved considerable development, making great contributions to the basic industry and machinery industry [4]. The casting process is used to obtain a part or workblank by pouring liquid metal into a cavity corresponding to the shape of the part, and then gradually cooling and solidifying it [5,6]. However, there are negative effects of the growing foundry industry, such as environment pollution [7,8,9] and casualties [10,11,12]. A severe explosion accident occurred in the Anshan Irion and Steel Group in China, killing 13 workers and injuring another 17 [10,12]. To improve the safe production of foundry operations and reduce the probability of accidents, it is necessary to strengthen foundry safety management. Strengthening the safety management of a foundry business will help to reduce unsafe behavior by employees. Assessment of safety management will allow workers to gain a clearer understanding of the safety level of a foundry business.

Frequently used safety assessment methods include fuzzy evaluation [13,14,15], grey relational analysis [16,17,18], neural network [19,20,21], and the cloud model [22,23,24]. To better evaluate various failure modes, a new fuzzy hybrid model to analyze failure modes and effects was proposed by Fattahi and Khalilzadeh [13], in which a fuzzy weight risk priority was considered for each failure. To search for environmentally friendly cutting fluids, Rapeti et al. [17] evaluated the performance of vegetable oil using grey relational analysis, and cost analysis for the application of different nanocutting fluids was done to assess the viability of these fluids in industry. George et al. [21] developed an artificial neural network for gasification process simulation based on extensive experimental data, and the results demonstrated this tool was useful for performance assessment of the gasification system. A trust evaluation method for clustered wireless sensor networks based on a cloud model was proposed by Zhang et al. [23], which converts qualitative and quantitative data from sensor nodes.

Of these assessment methods, the cloud model realized the uncertainty conversion between qualitative concepts and their corresponding quantitative values, as well as taking fuzziness and randomness into account [22,23,24]. Since the foundry business requires extensive production and safe production is affected by many factors, the relationship between some factors may not be explicit. Therefore, the cloud model is used to evaluate foundry businesses to protect employee health. In order to perform a safety assessment using the cloud model, the weights of the evaluation indicators must be known.

There are three main methods to obtain the weights of the evaluation indicators: subjective, objective, and integrated weight methods. The subjective weight methods include the analytic hierarchy process [25,26] and the Delphi method [27,28]. These subjective methods rely on subjective judgments from experts rather than actual data. This approach might take full advantage of learned knowledge, but the use of different experts could lead to different assessment results. The objective weight methods include principal component analysis [29,30], the entropy weight method [31,32], and the variation coefficient method [33,34]. These methods are mainly based on real data rather than expert judgments, taking advantage of the objectivity of real data, but the utilized data may not conform precisely to the actual situation. The integrated weight method combines subjective and objective weight methods into a single method, which combines expert judgments and real data [33]. For the preference coefficient of the integrated weight, there is no explicit computational method. To solve this issue, a new algorithm was proposed to determine the preference coefficient of the integrated weight based on the least square method [35] in this study.

After determining the appropriate safety level for a foundry business using the cloud model, corresponding safety measures should be adopted to protect employee health. Cause and effect reveals the causes of accidents and comprehensively in simple terms, allow various causes of accidents to be determined and analyzed [36,37]. However, the cause and effect does not determine the necessary safety measures corresponding to the identified risk factors. Layer of protection analysis (LOPA) is a semi-quantitative method to assess accident scenarios that analyzes the initiating event, consequences, and IPLs [10,38]. This study integrates these two methods for the first time into a cause and effect–LOPA method, which can identify the risk factors that may lead to accidents, and protect employee health using IPLs. The most representative research of occupational accidents analysis is safety barriers [39,40]. Safety barriers are an effective means against known risks, a way to prevent unwanted events from taking place and to protect against their consequences. The accident prevention measures that are adopted in this paper belong to safety barriers.

The purpose of this study was to build a composite safety assessment method for a foundry business using the cloud model and cause and effect–LOPA. To obtain the preference coefficient of the integrated weights more accurately, a new algorithm was proposed based on the least square method. Cause and effect were used to identify potential causes of accidents, and LOPA was used to identify safety measures that could protect against the identified risk factors.

## 2. Methods

### 2.1. Framework of the Proposed Safety Assessment Method

**Figure 1:**
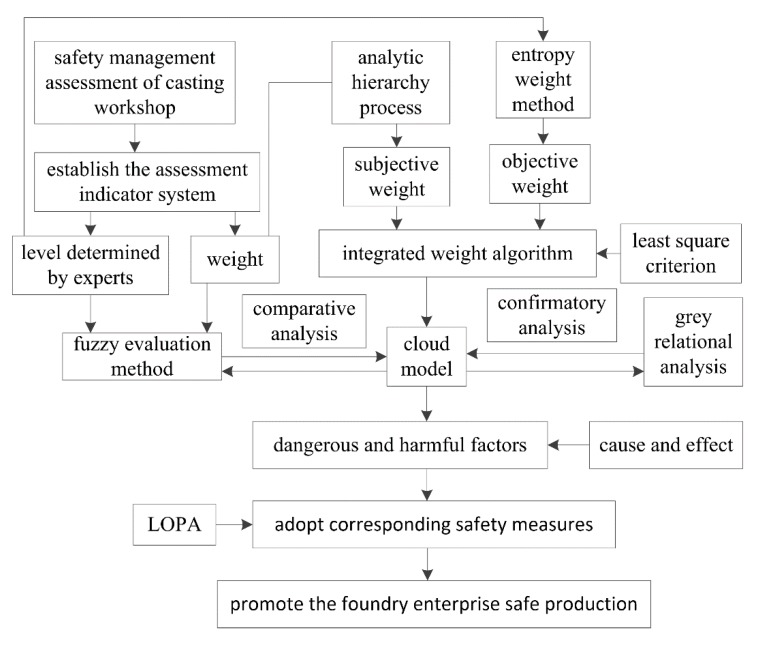
Framework of the proposed safety assessment method.

The framework of the proposed safety assessment method for a casting workshop is shown in Figure 1.

In order to determine the safety measures to be taken, the safety level of a casting workshop should be achieved first. A safety assessment is usually used to obtain the safety level of enterprises, and an assessment indicator system was established here, in which the sub-indicators mainly include educational training, safety input, dangerous and harmful factors control, hidden danger identification and security systems. After the subjective weights of the evaluated indicators were determined by analytic hierarchy process [25,26], and an appropriate safety situation was determined by experts for each evaluated indicator, the safety level of the casting workshop was preliminary assessed by the traditional fuzzy evaluation method [41]. However, the assessment process has two weaknesses: on the one hand, the fuzzy evaluation method does not reflect the randomness of assessment result; on the other hand, the subjective analytic hierarchy process does not take advantage of the objectivity of real data. The cloud model [22,23,24] was introduced to take randomness into consideration, the integrated weight method [33] was adopted, and a new algorithm was proposed to determine the preference coefficient of the integrated weight based on the least square method [35] in this study. To validate the cloud model and integrated weight algorithm for the safety assessment, grey relational analysis [16,17,18] was applied. Then, the causes and sub-causes of dangerous and harmful factors were identified by cause and effect analysis [36,37], and the causes mainly including dust, noise, toxic gas, mechanical injury, empyrosis and electric shock. In addition, 18 appropriate safety measures were identified using LOPA [10,38] to protect employee health and improve the safety level of the foundry business.

### 2.2. Fuzzy Evaluation Method

The fuzzy evaluation method is derived from fuzzy mathematics, and the level of the project is evaluated by fuzzy transformation and maximum membership degree [41]. The detailed procedure of the fuzzy evaluation method is described below.

The matrix *U* = {*u_j_*} represents the set of evaluated indicators, where 1≤j≤n and *n* is the number of indicators evaluated; the matrix *V* = {*v_i_*} is the set of evaluation levels, 1≤i≤m and *m* is the number of evaluation levels. The fuzzy evaluation matrix of the project is *R* = {*r_ij_*}, where *r_ij_* is the membership of the *j*th indicator evaluated at the *i*th evaluation level. The membership of the evaluated indicator is determined by the membership function [36].

The weight of indicator is evaluated to be $W=[w_{1},w_{2},\cdots ,w_{n}]\prime$.

The fuzzy evaluation result is obtained by the following equation.

(1) $$B=R\cdot W=[b_{1},b_{2},\cdots ,b_{m}]\prime$$

The safety level corresponding to the maximum *b_i_* is the final evaluation result according to the maximum membership principle.

### 2.3. Cloud Model

The cloud model is the specific uncertainty transformation between a qualitative concept and its corresponding quantitative value, in which the uncertainty transformation contains fuzziness and randomness, and the safety level of the casting workshop can be obtained by combining the qualitative and quantitative evaluation results [22,23,24]. A specific cloud model can be characterized by three numerical characteristics (*Ex, En, He*). The expectation *Ex* is the central value of the qualitative concept, the entropy *En* is the uncertain distribution of the qualitative concept, and the hyper entropy *He* is the fuzziness and randomness of *En*.

#### 2.3.1. Forward Cloud Algorithm

The forward cloud algorithm is used to generate as many cloud drops as needed based on the given numerical characteristics (*Ex, En, He*). It can be easily qualitatively analyzed through mapping the cloud model and the standard cloud models of indicators evaluated into one cloud image.

*Input*: The numerical characteristics (*Ex, En, He*) of the qualitative concept, and the number of cloud drops *n*.

*Output*: The position in the domain and membership of each cloud drop.

(1) Generate a normal random number *En′* with expectation *En* and standard deviation *He*.
(2) Generate a normal random number *x* with expectation *Ex* and standard deviation *En′*.
(3) Calculate $\mu \left(x\right)=e^{\frac{-{\left(x-Ex\right)}^{2}}{2{\left(En^{\prime }\right)}^{2}}}$
(4) Repeat procedures 1–3 until *n* cloud drops are created.

#### 2.3.2. Backward Cloud Algorithm

The backward cloud algorithm is used to calculate the numerical characteristics (*Ex*, *En*, *He*) from the given cloud drops.

*Input*. Cloud drops *x_i_* (*i* = 1, 2, …. *n*).

*Output*. Numerical characteristics (*Ex*, *En*, *He*) of the cloud drops.

(1) $Ex=\frac{1}{n}\sum_{i=1}^{n}x_{i}$
(2) $En=\sqrt{\frac{\pi }{2}}\times \frac{1}{n}\sum_{i=1}^{n}\left|x_{i}-Ex\right|$
(3) $He=\sqrt{\left|\frac{1}{n-1}{\sum_{i=1}^{n}\left(x_{i}-Ex\right)}^{2}-En^{2}\right|}$

#### 2.3.3. Standard Cloud Model

The standard cloud model is the standard for evaluating cloud models, and it is usually determined by the golden section method [22]. The standard cloud model is usually divided into an odd number, and is divided into five levels in this paper, including Safe *C_1_* (*Ex_1_, En_1_, He_1_*), Relatively safe *C_2_* (*Ex_2_, En_2_, He_2_*), Generally safe *C_3_* (*Ex_3_, En_3_, He_3_*), Relatively dangerous *C_4_* (*Ex_4_, En_4_, He_4_*), and Dangerous *C_5_* (*Ex_5_, En_5_, He_5_*). For the indicators evaluated with two unilateral constraints [*x_min_, x_max_*], the numerical characteristics of the standard cloud model can be calculated as follows based on the golden section method.

(2) $$Ex_{1}=x_{\max}$$

(3) $$Ex_{5}=x_{\min}$$

(4) $$Ex_{3}=\frac{x_{\max}+x_{\min}}{2}$$

(5) $$Ex_{2}=Ex_{3}+0.382\cdot \frac{x_{\max}+x_{\min}}{2}$$

(6) $$Ex_{4}=Ex_{3}-0.382\cdot \frac{x_{\max}+x_{\min}}{2}$$

(7) $$En_{2}=En_{4}=0.382\cdot \frac{x_{\max}-x_{\min}}{6}$$

(8) $$En_{3}=0.618\cdot En_{2}$$

(9) $$En_{1}=En_{5}=\frac{En_{2}}{0.618}$$

(10) $$He_{3}=k\cdot En_{3}$$

(11) $$He_{2}=He_{4}=\frac{He_{3}}{0.618}$$

(12) $$He_{1}=He_{5}=\frac{He_{2}}{0.618}$$

where the parameter *k* in Equation (10) may be changed based on the fuzziness and randomness of the indicators evaluated [24]. A larger *He*, as mentioned above, indicates greater randomness of assessment indicators; a smaller *He* suggests less randomness of the assessment indicators and randomness that is more easily lost [42]. Usually, *k* is no more than one third, and we set *k* = 0.1 in this treatment according to reference [22].

#### 2.3.4. Comprehensive Cloud Model

The cloud model of indicators evaluated is *C_i_* (*Ex_i_, En_i_, He_i_*) and the final comprehensive cloud model is *C* (*Ex, En, He*), in which *C_i_* is the fundamental cloud model of *C*. The comprehensive cloud model *C* can be computed as follows.

(13) $$Ex=\frac{\sum_{i=1}^{n}Ex_{i}En_{i}v_{i}}{\sum_{i=1}^{n}En_{i}v_{i}}$$

(14) $$En=\sum_{i=1}^{n}En_{i}v_{i}$$

(15) $$He=\frac{\sum_{i=1}^{n}He_{i}En_{i}v_{i}}{\sum_{i=1}^{n}En_{i}v_{i}}$$

where *v_i_* is the weight of the indicators evaluated and *n* is the number of indicators evaluated.

#### 2.3.5. Similarity between the Cloud Model and Standard Cloud Model

Similarity is used to determine the quantitative evaluation result of the cloud model. By calculating the similarity between the cloud model of the indicator evaluated and its corresponding standard cloud models, the specific quantitative evaluation result can be obtained.

The similarity between the cloud model and standard cloud model can be characterized as follows [22].

(16) $$\lambda_{j}=e^{-\frac{{\left(Ex-Ex_{j}\right)}^{2}}{2{\left(En_{j}\right)}^{2}}},$$

where *Ex* is the cloud model expectation of the indicator evaluated, *Ex_j_* is the entropy of the *j*th standard cloud model, and *En_j_* is the hyper entropy of the *j*th standard cloud model.

The level of the standard cloud model corresponding to the maximum similarity *λ_j_* is the quantitative evaluation result based on the maximum membership principle.

### 2.4. Grey Relational Analysis

Grey relational analysis is widely used in grey system theory [16,17,18], which is applied to calculate the grey relational degree among different evaluated indicators, and the safety level of the casting workshop can be achieved by grey relational degree. The detailed process of grey relational analysis is as follows.

If the original data matrix is *Y* = [*y_ij_*], the optimal index set is $D=[d_{1},d_{2},\cdots ,d_{n}]$, where *d_j_* is the optimal value of the *j*th evaluated indicator. For the larger the better indicator, *d_j_* is the maximum for the indicator.

The dimensionless nature of each indicator can be achieved as follows.

(17) $$z_{ij}=\frac{y_{ij}}{\max\{y_{ij}\}}\;j=1,2,\cdots ,n$$

After becoming dimensionless, the original data matrix can be transferred into *Z* = [*z_ij_*], and the optimal index set is transferred into $D^{*}=[d_{1}{}^{*},d_{2}{}^{*},\cdots ,d_{n}{}^{*}]$.

If the optimal index set D^*^ is the reference sequence and the matrix *Z* is the sequence compared, then the grey relational coefficient of the *j*th indicator evaluated for the *i*th evaluation level can be calculated as follows.

(18) $$\xi_{ij}=\frac{\underset{1\leq j\leq n}{\underset{1\leq i\leq m}{\min}}\left|d_{j}{}^{*}-z_{ij}\right|+\rho \underset{1\leq j\leq n}{\underset{1\leq i\leq m}{\max}}\left|d_{j}{}^{*}-z_{ij}\right|}{\left|d_{j}{}^{*}-z_{ij}\right|+\rho \underset{1\leq j\leq n}{\underset{1\leq i\leq m}{\max}}\left|d_{j}{}^{*}-z_{ij}\right|}$$

where ρ∈[0,1] is the resolution coefficient, and is usually set to ρ=0.5.

Additionally, if the weight of the indicator evaluated is $W={\left[w_{1},w_{2},\cdots ,w_{n}\right]}^{\prime }$ then the grey relational degree of the indicator evaluated can be obtained as follows.

(19) $$\tau_{i}=\sum_{j=1}^{n}\xi_{ij}\times w_{j}\;i=1,2,\cdots ,m$$

The larger the grey relational degree, the closer the evaluated indicator is to the optimal index set. Accordingly, the evaluation level of the project is confirmed.

### 2.5. Cause and Effect–LOPA

**Figure 2:**
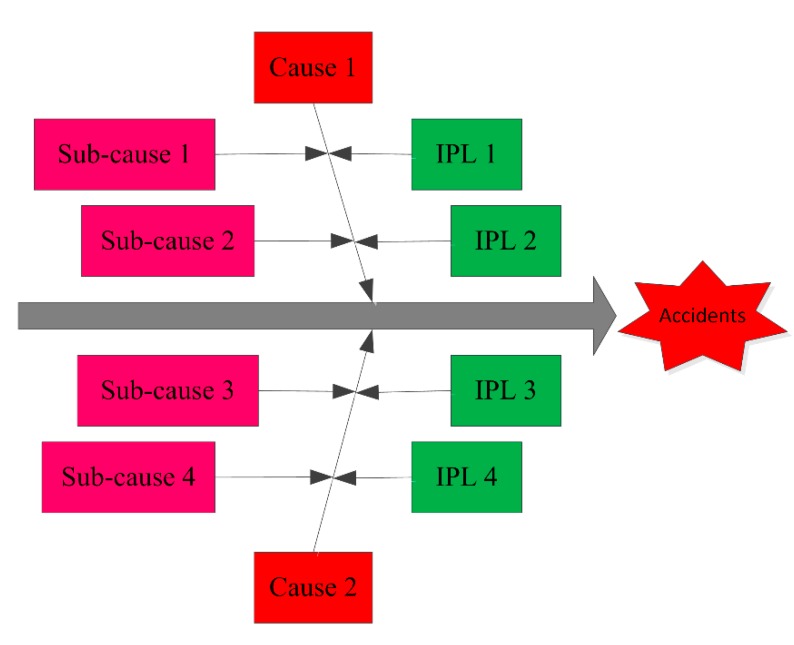
Cause and effect–LOPA diagram.

Once the safety level of the casting workshop is achieved, corresponding safety measures should be adopted. The cause and effect diagram clearly and comprehensively shows the causes of accidents in simple words, facilitating analysis [36,37]. LOPA is a semi-quantitative approach to assess accident scenarios that analyzes initiating events, consequences, and IPLs [10,38]. Cause and effect–LOPA identifies factors that may lead to accidents, and describes IPLs that could be applied to prevent accidents (Figure 2).

## 3. Results

### 3.1. Fuzzy Evaluation of Casting Workshop

A mechanical manufacturing business in Hunan, China was built in 1958, which covers an area of more than one point eight million square meters and has about five hundred workers. The registered capital of this mechanical manufacturing business is two billion *yuan*. The number of different types of equipment is more than 2800.

In recent years, this foundry business produced the first high performance driverless road roller in China, as well as the first environmentally friendly compacting machine for rubbish. Casting is a metal hot working process for producing components via mechanical manufacturing, which plays an important role in the national economy. Although it’s a service in social development, casualty accidents still occur. This study aims at adopting corresponding counter measures based on the safety evaluation result.

Analysis of the foundry site revealed that the factors affecting safety management of a casting workshop mainly include educational training, safety input, control of dangerous and harmful factors, hidden danger identification, and security systems. Therefore, the set of evaluated indicators is *U*={educational training(ET), safety input(SI), dangerous and harmful factors control(DHFC), hidden danger identification(HDI), security system(SS)}. The set of evaluation levels can be divided into *V*={Safe, Relatively safe, Generally safe, Relatively dangerous, Dangerous}.

After comparing the relative importance of evaluation indicators according to the analytic hierarchy process [25,26], the judgment matrix can be achieved as follows [43].

Based on reference [25,26], we can see that the degree of preference may reach up to 9 in extreme circumstances for different industries and different evaluation indicators. For the casting workshop of the foundry enterprise, there is not much difference among the importance of different evaluation indicators in Table 1. Taking ET and SI as an example, the scale of ET to SI is 2, indicating that the importance of ET is mildly (less than slightly) favored over SI. It should be noted that different weights of evaluation indicators can be achieved based on different degrees of preference. As mentioned in the Introduction, the weights achieved by the analytic hierarchy process mainly rely on subjective judgments from experts rather than actual data. Although the analytic hierarchy process might take full advantage of experts, different assessment results might be obtained from different experts.

The maximum eigenvalue was *λ_max_* = 5.1947, and the corresponding eigenvector was W = (0.2933, 0.5107, 0.387, 0.6738, 0.2223).

The consistency index was $CI=\frac{\lambda_{\max}-n}{n-1}=0.0487$, and the consistency ratio was $CR=\frac{CI}{RI}=\frac{0.0487}{1.12}=0.0435<0.1$, which indicates the judgment matrix was a consistent matrix [26].

The subjective weight can be calculated as follows after normalization.

*W_s_* = [0.141 0.245 0.185 0.322 0.107]′.

After experts vote on the level of each indicator evaluated, the evaluation matrix can be constructed by the vote ratio as follows [43].

$$R=\left[\begin{array}{l} 0.12\;\;0.09\;\;0.112\;0.133\;0.101\\ 0.201\;0.204\;0.347\;0.32\;\;0.217\\ 0.354\;0.403\;0.302\;0.317\;0.364\\ 0.207\;0.2\;0.209\;0.195\;0.211\\ 0.118\;0.103\;0.03\;\;0.035\;0.107 \end{array}\right]$$

For the evaluation matrix *R*, taking *ET* as an example, which means 12% of expert score 5, 20.1% of expert score 4, 35.4% of expert score 3, 20.7% of expert score 2 and 11.8% of expert score 1 for this indicator. The bigger the score, the higher the safety level of the casting workshop.

The fuzzy evaluation result can be obtained as follows, after application of Equation (1)

$$B=R\cdot W_{s}=\left[0.113\;0.269\;0.346\;0.202\;0.07\right]\prime$$

Therefore, the safety management evaluation result for the casting workshop was “Generally safe” based on the maximum membership principle.

### 3.2. Integrated Weight Determined by Least Square Method

The subjective weight method relies on the subjective judgments of experts, potentially resulting in variability in the assessment results. The objective weight method is based on actual data rather than expert judgments, but may not be exactly relevant to a given situation. The integrated weight method combines subjective and objective weight methods to include both expert judgments and data. The widely used integrated weight method is described as follows [33,44].

(20) $$w_{j}^{I}=\delta w_{j}^{S}+\left(1-\delta \right)w_{j}^{O}\;1\leq j\leq n$$

where, $w_{j}^{I}$, $w_{j}^{S}$ and $w_{j}^{O}$ indicate integrated, subjective, and objective weights, respectively; *n* is the number of indicators evaluated; δ∈[0,1] is the preference coefficient.

For the preference coefficient δ, there is no explicit computational method. Thus, in this study, a new algorithm was proposed to determine the preference coefficient based on the least square method [35] as follows.

The error sum of square among integrated, subjective, and objective weights of the evaluated indicator can be calculated as shown below.

(21) $${\sum_{j=1}^{n}\left(w_{j}^{I}-w_{j}^{S}\right)}^{2}+{\sum_{j=1}^{n}\left(w_{j}^{I}-w_{j}^{O}\right)}^{2}={\sum_{j=1}^{n}\left(\delta w_{j}^{S}-w_{j}^{S}+w_{j}^{O}-\delta w_{j}^{O}\right)}^{2}+{\sum_{j=1}^{n}\left(\delta w_{j}^{S}-\delta w_{j}^{O}\right)}^{2}$$

The least square method requires the least error sum of the square, so that Equation (21) achieves the minimum value.

Additionally, the method requires the derivation of the preference coefficient δ to allow Equation (20) to achieve the minimum value so that the derivative is 0, as shown in Equation (22).

(22) $$\left(2\delta -1\right){\sum_{j=1}^{n}\left(w_{j}^{S}-w_{j}^{O}\right)}^{2}=0$$

In Equation (22), the polynomial ${\sum_{j=1}^{n}\left(w_{j}^{S}-w_{j}^{O}\right)}^{2}\geq 0$. For Equation (22) to be correct, the polynomial (2δ−1) must be 0. Therefore, δ=0.5.

If we then input δ=0.5 into Equation (20), we obtain Equation (23)

(23) $$w_{j}^{I}=\frac{w_{j}^{S}+w_{j}^{O}}{2}$$

Although Equation (23) has the same form as references [33,44], it has more explicit physical significance in this study due to application of the least square method, that is, the error sum of the square among integrated, subjective and objective weights achieve the minimum value.

The objective weights of the evaluation indicators can be achieved by the entropy weight method [28,29] and the evaluation matrix R, giving results of W^O^ = [0.12 0.202 0.281 0.244 0.153]′. The findings results show that there is significant difference among different weights of evaluation indicators, such as the weight of DHFC is more than twice over ET. The objective entropy weight method is mainly based on real data rather than expert judgments, taking advantage of the objectivity of real data.

Therefore, the integrated weights of the indicator evaluated can be determined based on Equation (23), as: *W^I^* = [0.1305 0.2235 0.233 0.283 0.13]′.

### 3.3. Cloud Model Evaluation of Sub-indicators

If we set the numerical range of safety level as [1,5], then the corresponding standard cloud models can be achieved. Taking C_3_(*Ex*_3_*, En*_3_*, He*_3_) as an example, the expectation *Ex*_3_ can be calculated according to Equation (24) as follows.

(24) $$Ex_{3}=\frac{x_{\max}+x_{\min}}{2}=\frac{5+1}{2}=3$$

The entropy *En*_3_ can be calculated according to Equations (25) and (26) as follows.

(25) $$En_{2}=En_{4}=0.382\cdot \frac{x_{\max}-x_{\min}}{6}\approx 0.255$$

(26) $$En_{3}=0.618\cdot En_{2}=0.618\times 0.255\approx 0.158$$

The hyper entropy *He*_3_ can be calculated according to Equation (27) as follows.

(27) $$He_{3}=k\cdot En_{3}=0.1\times 0.158\approx 0.016$$

Then the standard cloud models of safety levels can be achieved in a similar way based on Equations (2)–(12), shown in Table 2.

For the evaluation matrix, assuming that one thousand experts are taking part in the vote. Taking ET as an example, 120 experts score 5, 201 experts score 4, 354 experts score 3, 207 experts score 2 and 118 experts score 1 for this indicator. The cloud model of ET can be obtained via a backward cloud algorithm, the expectation *Ex* can be calculated as follows.

(28) $$Ex=\frac{1}{n}\sum_{i=1}^{n}x_{i}=\frac{120\times 5+201\times 4+354\times 3+207\times 2+118\times 1}{1000}=2.998$$

The entropy *En* can be calculated based on the backward cloud algorithm as follows.

(29) $$En=\sqrt{\frac{\pi }{2}}\times \frac{1}{n}\sum_{i=1}^{n}\left|x_{i}-Ex\right|=\sqrt{\frac{3.14}{2}}\times \frac{1}{1000}\sum_{i=1}^{1000}\left|x_{i}-2.998\right|\approx 1.1085$$

The hyper entropy *He* can be calculated based on backward cloud algorithm as follows.

(30) $$He=\sqrt{\left|\frac{1}{n-1}{\sum_{i=1}^{n}\left(x_{i}-Ex\right)}^{2}-En^{2}\right|}=\sqrt{\left|\frac{1}{1000-1}{\sum_{i=1}^{1000}\left(x_{i}-2.998\right)}^{2}-1.1085^{2}\right|}\approx 0.364$$

The cloud models of other evaluation indicators can be achieved in a similar way, with the help of MATLAB software (The MathWorks Inc. Natick, MA, USA), shown in Table 3.

**Figure 3:**
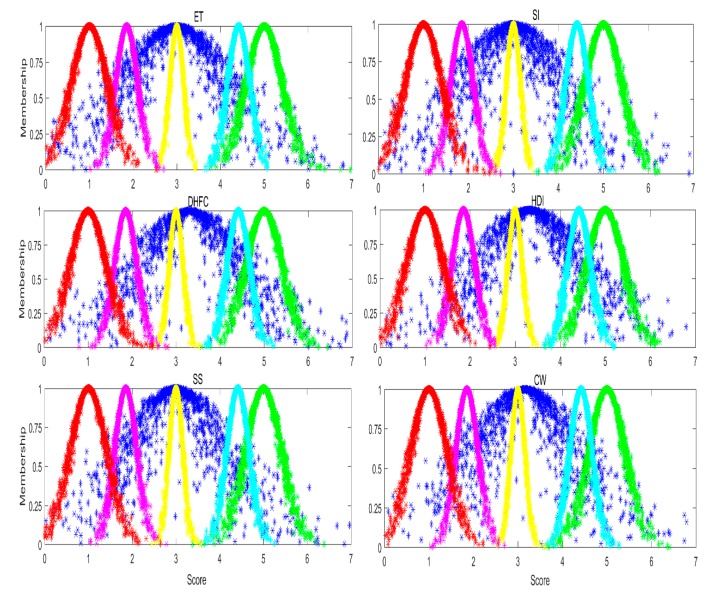
Comparison of indicator cloud models and corresponding standard cloud model. Red, pink, yellow, cyan and green standard cloud model indicate Dangerous, Relatively dangerous, Generally safe, Relatively safe and Safe levels, respectively. Cloud models of evaluation indicators marked in blue. All the cloud images consist of 1000 cloud drops.

The qualitative evaluation result can be obtained by mapping the cloud model, and its corresponding standard cloud models of ET into a cloud picture, shown in Figure 3.

As shown in Figure 3, the main cloud model of ET falls between the Relatively dangerous and Relatively safe standard cloud models, and the cloud drops of ET cloud model are most concentrated in the region of the Generally safe standard cloud model. Therefore, the qualitative evaluation result of ET was between Relatively dangerous and Relatively safe, and more inclined to Generally safe.

The assessment result of ET was the same in comparison against the expectation and corresponding safety levels (Table 2), which was in line with Xu et al.’s intuitive understanding that the assessment result of the cloud model was mainly based on the expectation of the evaluation indicator [45]. Safety level was worse when the expectation of the evaluation indicator was poor, which confirms that the introduced cloud model yields an accurate safety assessment. A qualitative assessment was obtained by comparing the cloud model of ET and its corresponding standard cloud models. The cloud drops of ET, as discussed above, are a good example of this, as most of the cloud drops fall between Relatively dangerous and Relatively safe, and are more inclined to Generally safe. The qualitative assessment result indicates that the safety level of ET was between Relatively dangerous and Relatively safe, and more inclined to Generally safe. Greater cloud model coverage area also indicates greater fuzziness in determining the corresponding safety level; in other words, the safety evaluation data were scattered across a very wide range and had large changes in safety levels. Safety indicators with greater cloud thickness also showed greater randomness; that is to say, the same safe score may have different membership degrees. For example, the membership degrees of cloud drops belonging to Relatively dangerous were from 0.3 to 0.8 in the case of the safety indicator of ET at score 2 (Figure 3).

From the above analysis, the qualitative evaluation result of ET is more likely to be Generally safe, indicating that the performance of ET is not very high, and corresponding safety measures should be adopted.

It is necessary to calculate the similarity between cloud model of ET and corresponding standard cloud models, to determine the specific safety level to which it belonged. Therefore, the similarities between this cloud model and corresponding standard cloud model can be obtained using Equation (16), shown in Table 4.

*λ*_3_ = 0.99992 was the maximum similarity of ET evaluation indicator, indicating the safe level of ET belonged to Generally safe. That is to say, the quantitative evaluation result of ET was Generally safe.

When determining the safety level of the evaluation indicator by the cloud model, the maximum similarity corresponding to the standard cloud model is the quantitative evaluation result based on the maximum membership principle. As shown in Table 4, not all the maximum similarities of evaluation indicators are close to 1, which reflected the uncertainty conversion between qualitative concepts and their corresponding quantitative values, and the uncertainty conversion containing fuzziness and randomness. Compared with the fuzzy evaluation method, although not all the maximum similarities of evaluation indicators are close to 1, the distinction degree of similarities is more remarkable (Table 4). Taking HDI as an example, although the maximum similarity is *λ*_3_ = 0.127, which is about 488 and 24 times over *λ*_1_ and *λ*_2_ respectively.

The qualitative evaluation result of ET was between Relatively dangerous and Relatively safe, and more inclined to Generally safe. The quantitative evaluation result of ET was Generally safe. Therefore, by combining the qualitative and quantitative evaluation results, the evaluation result of ET was Generally safe.

The evaluation results of other sub-indicators can be obtained in a similar way, and the evaluation results of SI, DHFC, HDI and SS were all Generally safe.

### 3.4. Cloud Model Evaluation of Casting Workshop

The comprehensive cloud model of the casting workshop (CW) can be achieved via Equations (13)–(15), shown in Table 3.

The qualitative evaluation result can be obtained by mapping the cloud model of the CW, and the corresponding standard cloud model into a cloud image, shown in Figure 3.

As shown in Figure 3, the main cloud model of CW falls between the Relatively dangerous and Relatively safe standard cloud models, with the cloud drops of the CW cloud model being most concentrated in the region of the Generally safe standard cloud model. Therefore, the qualitative evaluation result of CW was between Relatively dangerous and Relatively safe, and more inclined to Generally safe.

It is necessary to calculate the similarity between cloud model of CW and the corresponding standard cloud models, to determine the specific safe level to which it belonged. Therefore, the similarities between this cloud model and the corresponding standard cloud model can be obtained using Equation (16), shown in Table 4.

*λ*_3_ = 0.60196 was the maximum similarity of CW evaluation indicator, indicating the safe level of CW belonged to Generally safe. That is to say, the quantitative evaluation result of CW was Generally safe.

The qualitative evaluation result of CW was between Relatively dangerous and Relatively safe, and more inclined to Generally safe. The quantitative evaluation result of CW was Generally safe. Therefore, by combining the qualitative and quantitative evaluation results, the evaluation result of CW was Generally safe.

### 3.5. Comparison by Grey Relational Analysis

To validate the integrated weight algorithm proposed in this study, we next used grey relational analysis to compare the safety assessment results [16,17,18] with the cloud model. The process of grey relational analysis is illustrated below.

The dimensionless matrix of the evaluated indicator can be achieved as follows based on Equation (31).

(31) $$Z=\left[\begin{array}{l} 0.339\;0.223\;0.323\;0.416\;0.277\\ 0.568\;0.506\;1\;1\;0.596\\ \;1\;1\;0.87\;0.991\;1\\ 0.585\;0.496\;0.602\;0.609\;0.58\\ 0.333\;0.256\;0.086\;0.109\;0.294 \end{array}\right]$$

The optimal index set was transferred into D* = [11111].

The grey relational coefficient was achieved based on Equation (32).

(32) $$\xi =\left[\begin{array}{l} 0.409\;0.37\;0.403\;0.439\;0.387\\ 0.514\;0.481\;1\;1\;0.531\\ \;1\;1\;0.779\;0.98\;1\\ 0.524\;0.476\;0.535\;0.539\;0.521\\ 0.407\;0.38\;\;0.333\;0.339\;0.393 \end{array}\right]$$

If the integrated weight of the indicator evaluated is *W^I^* = [0.133 0.2315 0.231 0.2745 0.13]′, then, the grey relational degree of casting workshop can be determined as below, according to Equation (33).

(33) $$\tau =\xi \times W^{I}=\left[0.404\;0.754\;0.943\;0.519\;0.363\right]\prime$$

The evaluation level for the casting workshop was “Generally safe” according to the grey relational degree.

The assessment result determined by grey relational analysis was the same as that obtained by the cloud model, in which indicator weights were determined using the revised integrated weight algorithm. Thus, the integrated weight method proposed and cloud model adopted in this study are feasible.

### 3.6. Cause and Effect–LOPA of Dangerous and Harmful Factors

**Figure 4:**
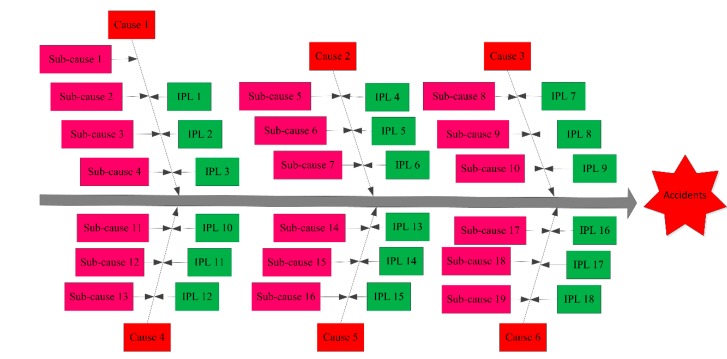
Cause and effect–LOPA of dangerous and harmful factors.

The safety management assessment indicated conditions are likely to be Generally safe based on the above analysis. Accidents are probable in the foundry workplace, due to dangerous and potentially harmful conditions. Controlling potentially dangerous factors will help to improve the safe operation of the foundry. To do this, cause and effect–LOPA was applied to identify dangerous and harmful factors that contribute to accidents, as shown in Figure 4.

As shown in Figure 4, the causes that may lead to accidents in the casting workshop mainly including dust, noise, toxic gas, mechanical injury, empyrosis and electric shock, and each cause contains several sub-causes. Taking dust as an example, whose sub-causes are sand mixing, modeling, shakeout and fettling, that is, from sub-causes 1 to 4. In other words, the dust in the casting workshop is likely to be caused by sub-causes 1–4. Similarly, sub-causes of noise are from 5 to 7, sub-causes of toxic gas are from 8 to 10, sub-causes of mechanical injury are from 11 to 13, sub-causes of empyrosis are from 14 to 16, and sub-causes of electric shock are from 17 to 19. The causes and sub-causes that may lead to accidents in the casting workshop are shown in Table 5.

In Figure 4, to prevent accidents in the casting workshop and protect employee health, 18 IPLs should be adopted. Taking dust as an example, whose IPLs are wearing a mask, wet working and dust removal by ventilation, that is, from IPLs 1 to 3. In other words, the dust in the casting workshop can be eliminated by IPLs 1–3. Similarly, IPLs of noise are from 4 to 6, IPLs of toxic gas are from 7 to 9, IPLs of mechanical injury are from 10 to 12, IPLs of empyrosis are from 13 to 15, and IPLs of electric shock are from 16 to 18. The IPLs that can be adopted to prevent accidents and protect employee health are shown in Table 6.

In this cause and effect–LOPA, foundry accidents were attributed to 6 causes and 19 sub-causes, and foundry accidents can be prevented by 18 IPLs. Causes 1 to 6 are all risk factors, which can lead to accidents in the casting workshop. Causes 1 and 2 may result in organ failure of the body and belong to low risk factors; Causes 3 to 6 can lead to serious casualties and belong to high risk factors. If the IPLs proposed in this study were not carried out, accidents may occur in the casting workshop. The safety level of the foundry can be improved by taking steps based on the cause and effect–LOPA identification of dangerous and harmful factors.

From the standpoint of safety management, to improve safe production in the casting workshop, management measures should also be adopted. First, improve the rules and regulations for the casting workshop. Second, strengthen the safety training. Third, the identification of dangerous and harmful factors and elimination of accident potential.

## 4. Discussion

As a traditional safety assessment method, the fuzzy evaluation method can deal with the fuzziness during conversion [14]. The safe production of a foundry business is affected by many factors, and the relationships between some factors may be uncertainty and even randomness. In this case, the fuzzy evaluation method is not suitable for determining the safety level of the foundry enterprise. The cloud model realized the uncertainty conversion between qualitative concepts and their corresponding quantitative values, as well as taking fuzziness and randomness into account [22,23,24]. By combining the qualitative and quantitative evaluation results, the evaluation result of the cloud model can be achieved. Therefore, the cloud model was introduced in the safety assessment of the foundry enterprise, and the validity of the cloud model evaluation was validated by grey relational analysis [16,17,18].

In order to perform a safety assessment of the foundry enterprise, the weights of the evaluation indicators must be known. For the preference coefficient of the integrated weight, there is no explicit computational method. To solve this issue, a new algorithm was proposed to determine the preference coefficient of the integrated weight based on the least square method [35] in this study. The integrated weight algorithm proposed in this study has more explicit physical significance due to application of the least square method, that is, the error sum of the square among integrated, subjective and objective weights achieves the minimum value.

Once the safety level for the foundry enterprise has been determined, corresponding safety measures should be adopted to protect employee health. Fault tree analysis [46,47], the traditional accident analysis method, can identify the causes of an accident, but cannot perform detailed analysis of identified risk factors. The cause and effect analysis method can identify the causes of the identified risk factors [36,37], and LOPA was applied to identify appropriate safety measures corresponding to the identified risk factors [10,38]. This study integrates these two methods into a cause and effect–LOPA method, which can identify in advance factors that may lead to accidents, and protect employee health using IPLs. The most representative research of occupational accidents analysis is safety barriers [39,40]. Safety barriers are an effective means against known risks, a way to prevent unwanted events from taking place and to protect against their consequences. The accident prevention measures that are adopted in this paper belong to safety barriers.

To simplify the discussion, the parameter *k* in Equation (10) was chosen as only 0.1. Future research should focus on the influence of this parameter on the safety evaluation result.

## 5. Conclusions

A composite safety assessment model for a casting workshop based on the cloud model and cause and effect–LOPA was proposed in this study to protect employee health. The main conclusions are shown below.

After the weights of evaluation indicators were determined using the subjective analytic hierarchy process and objective entropy weight method respectively, a new integrated weight algorithm was proposed based on the least square method. The integrated weight determined by the least square method has more explicit physical significance in this study, that is, the error sum of the square among integrated, subjective and objective weights achieves the minimum value.

The safety level of the casting workshop was Generally safe based on the qualitative and quantitative analysis of the cloud model, which realized the uncertainty conversion between qualitative concepts and their corresponding quantitative values, as well as taking fuzziness and randomness into account. The validity of the cloud model evaluation was validated by grey relational analysis.

The potentially dangerous and harmful factors were analyzed using cause and effect–LOPA, identifying 6 causes and 19 sub-causes that may lead to accidents and 18 IPLs that could prevent accidents in a casting workshop. The safety level of this foundry could thus be improved by applying the cause and effect–LOPA of potential risk factors.

## Figures and Tables

**Table 1:** Judgment matrix of evaluation indicator of a casting workshop.

Evaluation Indicator	*ET*	*SI*	*DHFC*	*HDI*	*SS*
*ET*	1	1/2	1/2	1/2	2
*SI*	2	1	2	1/2	2
*DHFC*	2	1/2	1	1/2	2
*HDI*	2	2	2	1	2
*SS*	1/2	1/2	1/2	1/2	1

**Table 2:** Score and standard cloud model of safety evaluation level.

Level	Score	Standard Cloud Model
Safe	5	C_1_(5,0.413,0.042)
Relatively safe	4	C_2_(4.146,0.255,0.026)
Generally safe	3	C_3_(3,0.158,0.016)
Relatively dangerous	2	C_4_(1.854,0.255,0.026)
Dangerous	1	C_5_(1,0.413,0.042)

**Table 3:** Cloud model of evaluation indicator.

Indicator	Cloud Model
ET	(2.998,1.1085,0.364)
SI	(2.978,1.0007,0.4186)
DHFC	(3.302,1.0835,0.3745)
HDI	(3.321,1.1041,0.3661)
SS	(2.994,1.0603,0.3702)
CW	(3.1592,1.0711,0.3793)

**Table 4:** Similarities between the cloud model of evaluation indicators and the corresponding standard cloud model.

Indicator	Similarity				
	*λ*_1_	*λ*_2_	*λ*_3_	*λ*_4_	*λ*_5_
ET	0	0.00004	0.99992	0.00004	0
SI	0	0.00003	0.99035	0.00006	0.00001
DHFC	0.00021	0.00418	0.16097	0	0
HDI	0.00026	0.00534	0.127	0	0
SS	0	0.00004	0.99928	0.00005	0
CW	0.00005	0.00056	0.60196	0	0

**Table 5:** Detailed description of causes.

Cause	Description	Cause	Description
Cause 1	Dust	Sub-cause 8	Alloy melting and casting
Cause 2	Noise	Sub-cause 9	Welding operation
Cause 3	Toxic gas	Sub-cause 10	Swabbing
Cause 4	Mechanical injury	Sub-cause 11	Unsafe condition of equipment
Cause 5	Empyrosis	Sub-cause 12	Unsafe behavior of human
Cause 6	Electric shock	Sub-cause 13	Safe distance is not sufficient
Sub-cause 1	Sand mixing	Sub-cause 14	Molten metal spatter
Sub-cause 2	Modelling	Sub-cause 15	Contact with high temperature smelter
Sub-cause 3	Shakeout	Sub-cause 16	Contact with uncooled casting and core
Sub-cause 4	Fettling	Sub-cause 17	Electrical equipment is defective
Sub-cause 5	Shakeout finishing	Sub-cause 18	Insulated wire aging
Sub-cause 6	Vibration modelling	Sub-cause 19	Safe voltage not used
Sub-cause 7	Air blower working		

**Table 6:** Detailed description of IPLs.

IPL	Description	IPL	Description
IPL 1	Wearing a mask	IPL 10	Rationally plan equipment installation location
IPL 2	Wet working	IPL 11	Isolating work areas and non-work areas with barriers
IPL 3	Dust removal by ventilation	IPL 12	Employees must abide by operating regulation
IPL 4	Wearing earplugs	IPL 13	Wearing high temperature protective equipment
IPL 5	Set up sound proof wall	IPL 14	Isolation of high temperature work area
IPL 6	Equipment with shock absorber	IPL 15	Alert when transporting molten metal
IPL 7	Strengthening ventilation	IPL 16	Design of electrical equipment to meet safety criterion
IPL 8	Using environment friendly coating	IPL 17	Establish and improve the operating guidelines for electrical equipment
IPL 9	Install air cleaning unit	IPL 18	Set a warning mark
